# Measures of fidelity of delivery and engagement in self-management interventions: A systematic review of measures

**DOI:** 10.1177/17407745221118555

**Published:** 2022-08-26

**Authors:** Tasmin A Rookes, Anette Schrag, Kate Walters, Megan Armstrong

**Affiliations:** 1 Centre for Ageing Population Studies, Primary Care and Population Health, University College London, London, UK; 2 Department of Clinical and Movement Neurosciences, Institute of Neurology, University College London, London, UK

**Keywords:** Self-management, fidelity, engagement, intervention delivery

## Abstract

**Background/Aims:**

Self-management interventions are increasingly being developed and researched to improve long-term condition outcomes. To understand and interpret findings, it is essential that fidelity of intervention delivery and participant engagement are measured and reported. Before developing fidelity checklists to assess treatment fidelity of interventions, current recommendations suggest that a synthesis of fidelity measures reported in the literature is completed. Therefore, here we aim to identify what the current measures of fidelity of intervention delivery and engagement for self-management interventions for long-term conditions are and whether there is treatment fidelity.

**Methods:**

Four databases (MEDLINE, PubMed, CINAHL Plus and ScienceDirect) and the journal implementation science were systematically searched to identify published reports from inception to December 2020 for experimental studies measuring fidelity of intervention delivery and/or participant engagement in self-management interventions for long-term conditions. Data on fidelity of delivery and engagement measures and the findings were extracted and synthesised.

**Results:**

Thirty-nine articles were identified as eligible, with 25 studies measuring fidelity of delivery, 19 reporting engagement and 5 measuring both. For fidelity of delivery, measures included structured checklists, participant completed measures and researcher observations/notes. These were completed by researchers, participants and intervention leaders. Often there was little information around the development of these measures, particularly when the measure had been developed by the researchers, rather than building on others work. Eighteen of 25 studies reported there was fidelity of intervention delivery. For engagement, measures included data analytics, participant completed measures and researcher observations. Ten out of 19 studies reported participants were engaged with the intervention.

**Conclusion:**

In complex self-management interventions, it is essential to assess whether treatment fidelity of each core component of interventions is delivered, as outlined in the protocol, to understand which components are having an effect. Treatment fidelity checklists comparing what was planned to be delivered, with what was delivered should be developed with pre-defined cut-offs for when fidelity has been achieved. Similarly, when measuring engagement, while data analytics continue to rise with the increase in digital interventions, clear cut-offs for participant use and content engaged with to be considered an engagement participant need to be pre-determined.

## Background

In the United Kingdom, it is estimated that 30% of the population have a long-term condition (LTC), which rises to 58% in those over 60 and this prevalence is increasing.^1^ The cost to the National Health Service was assessed in 2013 to be 70% of health and social care spending, which is likely to be higher now. With increasing prevalence and co-morbidities, which are often associated with depression and anxiety,^2^ the impact to society and the individual is of great concern. To tackle this, proactive self-management interventions have been developed to help these populations to manage the symptoms of their conditions themselves. Self-management involves the tasks a person must undertake to manage their LTC(s), covering three areas of management: medical management, emotional management and behaviour change through problem-solving and goal setting.^3,4^ There has been growing evidence that complex self-management interventions, with multiple active components – for example, education, exercise, self-monitoring, and independence – can help improve clinical outcomes, such as quality of life, and pain, in a range of LTCs.^5,6^

With the growing self-management literature, there has also been an increase in ambiguous findings on the effectiveness of these interventions. In a systematic review of self-management interventions in people with Parkinson’s, only 4/36 studies showed a significant improvement for quality of life in the intervention arm.^7^ To understand any lack of effect following a trial, a fidelity assessment is needed to determine if the intervention was delivered as planned in the protocol. This review will focus on two subcomponents of a fidelity assessment: fidelity of intervention delivery and fidelity of engagement. Fidelity of treatment delivery refers to the extent to which the intervention is delivered as expected, how much of the intervention is received and the enactment of the intervention by participants.^8^ Fidelity of participant engagement refers to how participants engage with the intervention content, including their understanding, ability to perform the skills needed and if these skills are then used in daily life.^8,9^ Having this information enables other researchers to infer whether the lack of effect is due to poor implementation or an ineffective intervention. Without this, potentially effective interventions could be disregarded due to poor implementation, resulting in a type II error.^8^

Measuring fidelity of delivery and engagement is particularly important in complex interventions to understand which components are required to result in a positive effect on outcomes.^10^ This ensures that when interventions are implemented, these core components are emphasised, so effects seen in trials are translated into clinical care. However, in the Walton et al.^9^ review of complex health behaviour change interventions, only 36% measured fidelity and engagement; the review by Pigott et al.^7^ found none of the included studies conducted a formal fidelity assessment, and reviewing implementation fidelity for self-management for osteoarthritis, Toomey et al.^11^ found very low fidelity reporting, with only 1/22 studies achieving acceptable reporting levels.

Choosing which aspect of fidelity is measured, how the chosen measure is developed and why are also often not reported or justified.^9^ Developing reliable measures for fidelity of delivery and engagement for trials can be a challenge, due to the uniqueness of each intervention, but is essential to interpret intervention effectiveness reliably. One review found that the measure used to assess fidelity was not reported in over half of cases for smoking cessation interventions.^12^ The Medical Research Council guidance proposes that mixed-methods evaluations, combining fidelity checklists with another measure, provide the greatest confidence in conclusions about effectiveness, by overcoming limitations of individual measures and gaining both a top level and detailed understanding of factors influencing fidelity.^13^

To standardise the process for developing fidelity checklists and measures, Walton et al.^10^ developed a systematic five-step guide, which can be applied to a variety of complex health interventions. The first step involves conducting a review of previously developed fidelity measures and checklists within the intervention group being investigated, to support decision-making for what to include in the current intervention checklists. Step 2 involves analysing the intervention components and developing a framework with the intervention content. In Steps 3, 4 and 5, the fidelity checklists are developed, refined and piloted.

When searching the self-management literature, although 969 unique self-management Randomised Controlled Trials (RCTs) were identified in 2014 when developing the PRISMS taxonomy^14^ and many more conducted since, no systematic review of fidelity measures for self-management interventions has been conducted. The purpose of this review was not to quantify how many self-management interventions assess fidelity, instead, in line with Step 1 of the Walton^10^ five-step guide and to develop the fidelity checklist for an RCT investigating the effectiveness and implementation of a self-management toolkit for people with Parkinson’s, we conducted this systematic review to identify previous fidelity measures used in this literature.

The aims were (1) to identify the measures used to assess the fidelity of intervention delivery and engagement with complex self-management interventions in people with LTCs and (2) to explore whether self-management interventions had treatment fidelity and were therefore delivered and engaged with as expected, according to the protocol.

## Methods

This review was conducted in accordance with PRISMA guidelines (Supplementary Table 1) and registered on PROSPERO (ID: CRD42020223129).

### Search strategy

The databases MEDLINE, PubMed, PsycINFO, CINAHL Plus and ScienceDirect and the journal Implementation Science were systematically searched from inception to December 2020. MeSH and CINAHL subject headings were used to guide the search term list for each database platform and for OVID example see Supplementary Figure 1. The database search articles were exported into EndNote and duplicates removed. Titles and abstracts were independently screened, by two reviewers (T.A.R. and M.A.), against the inclusion criteria. Any discrepancies were discussed between the two reviewers to determine if they were eligible for data extraction.

### Inclusion and exclusion criteria

#### Inclusion criteria

Papers were included if a self-management intervention with multiple components was aimed at adults, over 18 years old, with a clinical diagnosis of a LTC. Researchers had to report the findings of at least a quantitative measure of the fidelity of intervention delivery or participant engagement. Trial designs could be RCTs or quasi-experimental.

#### Exclusion criteria

Papers were excluded if they report (1) interventions that do not include human participants; (2) educational interventions with no tools for participants to implement learning; (3) interventions for non-clinical populations; (4) interventions delivered to carers or healthcare professionals; (5) engagement as number of sessions attended only (attendance); (6) ongoing studies; or (7) are only published as an abstract.

### Data extraction and analysis

Study characteristics, fidelity and engagement were extracted by T.A.R. and checked by M.A. for completeness. For all studies, we extracted trial authors, country, year of publication, intervention description and components, disease area, population size and statistical significance of the primary outcome. We extracted information on fidelity and/or engagement such as, what was measured, type of measure, who completed the measure, development of measure, sample, analysis method and summary of findings.

As measuring fidelity is unique to each intervention and study, a meta-analysis was not appropriate. The findings were synthesised narratively, describing the types of measures identified. As this review is examining measures of fidelity, rather than trial findings on effectiveness, a formal quality assessment was not undertaken.

## Results

**Figure 1.**
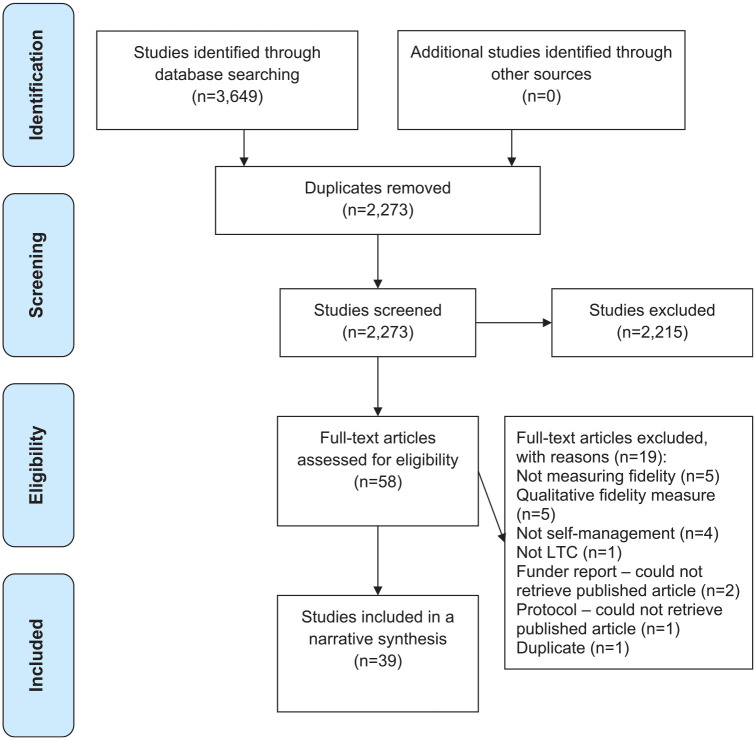
PRISMA systematic review flow diagram.

After database searching and duplicates were removed, 2276 titles and abstracts were identified for screening. Fifty-three full-text articles were screened for eligibility, of which 39 were included in the synthesis (see Figure 1 PRISMA flow diagram and Supplementary Figure 2 reference list of eligible articles).

### Study characteristics

Thirty studies (77%) were RCTs and nine (23%) used non-randomised designs. Nineteen (49%) were delivered face-to-face, seven (18%) delivered online, one (3%) on the telephone, three (8%) used an app and nine (23%) used some combination of the above. Seventeen individual LTCs were targeted in thirty-four studies, including diabetes, stroke, epilepsy, psychosis and cancer, with the other five looking at a combination of physical or mental health conditions. The oldest included study was completed in 2002 with a steady increase in the number of studies being reported per year over the past 20 years. Twenty (51%) of the studies were conducted in the last 4 years and almost 80% conducted in the last 6 years. Seven studies were conducted in Australia, four in the Netherlands, eleven in the United Kingdom and thirteen in the United States. The remaining four were in Ireland, Switzerland, Sweden and Singapore.

Components of the interventions included lifestyle management, education, goal setting, action planning, problem-solving, exercise, recovery and feedback. A full list of the included studies and their characteristics can be seen in Supplementary Table 2.

### Measuring fidelity of delivery and engagement

Twenty-five (64%) reported fidelity of intervention delivery and 19 (49%) reported participant engagement with the intervention. Of these, five (13%) measured both, 20 (51%) assessed fidelity of delivery only and 14 (36%) assessed engagement only. Full details of the fidelity of delivery and engagement methods, measures and results can be seen in Supplementary Tables 3 and 4, respectively.

### Fidelity of delivery

#### Types of measure

Assessing fidelity of intervention delivery was grouped into four main categories. Most used a structured checklist to compare what was delivered against the protocol (n=18; 72%). Others used participant completed questionnaires developed by the research team (n=6; 24%), researcher observation or notes from sessions (n=5; 20%) and online data analytics (n=1; 4%). In five (20%) studies, multiple measures were used, often combining a checklist with participant completed questionnaires.

#### Measure details

The structured checklists were often developed by the researchers based on their protocol (n=12; 48%), with little to no information surrounding the development process. In the studies that did describe the development process (n=6; 24%), this involved conducting a systematic review or piloting the checklist in a feasibility study before implementing in the RCT. Three studies (12%) used existing fidelity frameworks, with two of these studies comparing different frameworks for assessing fidelity. The frameworks were as follows: the RE-AIM framework aiming to increase attention on essential programme elements for validity and implementation, focusing on Reach, Effectiveness, Adoption, Implementation and Maintenance;^15^ PIPE framework assesses the public health impact of interventions, focusing on Penetration, Implementation, Participation and Effectiveness;^16^ DESMOND Observation Tool measures facilitator versus participant talk time^17^ and STEPWISE Core Facilitator Behavioural Observation Sheet assessing behaviour change, planning and goal setting against behaviour domains.^18^

Measures completed by participants used Likert-type scales to gain participants opinion on implementation of the intervention. In three studies, how these were developed was unclear. In one study, the researchers stated they developed it themselves without any description and the final one used the Health Education Impact Questionnaire.^19^ When researcher/facilitator notes were used, often less information was provided regarding what was collected and what it was compared with, making analysis and interpretation difficult. Non-digital measures were completed by either the researcher (n=12; 48%), intervention leader (n=9; 36%) and/or participant (n=5; 20%). In three studies (12%), it was unclear who completed the measures.

#### Sampling and analysis

For two (8%) studies, they sampled all participants, including the control, 13 (52%) measured fidelity of delivery for all participants in the intervention arm, eight (32%) randomly selected a sub-sample of the intervention arm and the sample was unclear in three (12%) studies. One study used different samples for the two measures. Descriptive summary statistics were used in 21 (84%) of the studies, statistical tests were used in four (16%) studies, such as reliability of measures (kappa) correlates between sites/staff and fidelity, and comparing participant progress between groups, and the analysis method was unclear in two (8%) studies.

#### Findings

Of the 25 studies measuring fidelity, researchers reported that their threshold for fidelity of intervention delivery was met in 18 of these studies (72%). In six of the studies (24%), the researchers reported no fidelity of intervention delivery, and in one study (4%), it was unclear. Of the six studies where the researchers found there was no fidelity, four studies used frameworks and checklists completed by researchers, one was a participant self-completed measure of progress towards goals and the other a facilitator consultation log. The study with unclear findings used facilitator reflection notes. Reporting of fidelity of delivery through researcher-completed fidelity checklists was often determined by reporting the percentage of checklist components completed as expected. For participant completed, these were often on a scale from not completed to completed. Despite researchers reporting these results and stating whether there was or was not fidelity, it was never reported what the threshold was and/or how this was determined. Reported levels of ‘high fidelity of delivery’ varied across articles, between 60% and 98% of checklist components completed.

### Engagement

#### Types of measure

For measuring engagement, three broad categories were identified. Data analytics were most frequently used (even though only available in digital interventions) (n=11; 58%), followed by participant completed measures (n=5; 26%) and researchers observing engagement (n=4; 21%). In just one (5%) study, multiple measures were used, combining data analytics and participant completed questionnaires.

#### Measure details

Digital measures included use over time (n=6; 32%), content engaged with (n=6; 32%) and uptake of referral numbers in General Practices (n=1; 5%). Participant completed measures largely covered goal setting and implementation (n=3; 16%), with adherence, enactment, homework completion and content engaged with each assessed once (5%). Researcher observations mainly included active participation in sessions (n=3; 16%), with one study measuring goal setting and implementation (5%). The digital measures were built into the app or website and automatically collected (n=11; 58%). For the other measures, three (16%) were developed from other studies or scales, two (11%) were developed by the researchers, with limited to no information on this process, and the development was unclear for five (26%) measures. Those that were unclear tended to be participant completed Likert-type scale measures or researcher observations. The scales developed from previous studies were: the FITT Index assessing the data analytics of Frequency, Intensity, Time, and Type for digital interventions;^20^ the Working Alliance Inventory – Short Revised measuring agreement on self-management tasks, goals, and bond between the interventionist and the patient^21^ and one was not stated.

#### Sampling and analysis

Four (21%) studies sampled all participants, 12 (63%) included just those receiving the intervention and four (21%) used a sub-sample of the main studies sample, with no studies having an unclear sample. One study used different samples for two different measures. Eighteen (95%) studies used descriptive summary statistics to analyse the engagement measures. Formal statistical tests, such as participant predictors of engagement and engagement associated with clinical outcomes, were used in four (21%) studies.

#### Findings

Of the 19 studies measuring engagement, in 10 (53%) researchers reported that their threshold for engagement was met. In six studies (32%), researchers reported that participants were not engagement, and in three studies (16%), it was unclear. Of those that found no engagement four measures were data analytics and two were participant completed measures. All three measures with unclear results were data analytic measures, often stating engagement findings, but not putting them into context regarding if this was sufficient or not.

## Discussion

This systematic review identified and synthesised research exploring fidelity of intervention delivery and engagement in complex self-management interventions. Twenty-five of the included studies reported fidelity of intervention delivery and 19 reported engagement, with five studies measuring both. To measure fidelity of delivery, most used a structured checklist developed from and compared against the protocol. Other measures included researcher observations and notes, participant completed measures and data analytics, and were either completed by the researcher, intervention leader or participants. Just under three quarters of studies reporting intervention fidelity found that it had been delivered as expected. To measure engagement, data analytics was used most frequently due to the increase in digital interventions, followed by participant completed measures and researcher observations. Just over half found that participants were engaged with the intervention. Over half of the included studies were conducted in the last 4 years and almost 80% conducted in the last 6 years, highlighting the increase in both self-management research and fidelity assessments of these interventions.

### Results in context

#### Fidelity of delivery

As seen in other systematic reviews exploring fidelity of intervention delivery, there was heterogeneity in the type of measure used, what was measured and who it was completed by.^22,23^ Using a less standardised approach, such as intervention leaders’ notes or researcher observation could result in biased outcomes. There is a chance that the data collected is less accurate or harder to analyse due to the lack of structure making outcomes difficult to interpret. Alternatively, intervention leaders may introduce measurement bias and rate things as being completed as intended in the protocol, even though they were not.^24^ A more robust way to assess fidelity of intervention delivery is to develop researcher-completed structured checklists, independently comparing what was conducted to what was stated in the intervention protocol, based on current evidence and guidelines, pilot them in a feasibility study and adapt them before applying to an RCT.^9,25^

Although structured checklists compared to the protocol are likely to be the most reliable and valid method to measure fidelity of delivery, it is important that authors provide more detail around the development and content of these checklists and how they can be applied, so they can be scrutinised and/or used as a basis for future research.^24,26^ In this review, fidelity of intervention was confirmed by researchers if checklists matched between 60% and 98%, but no papers pre-defined their cut-off. By not pre-defining the checklist threshold for fidelity to be confirmed, researchers could be interpreting their findings in a more positive light to emphasise the quality of their work.

#### Engagement

Using data analytics to measure engagement could produce less biased results due to the objective nature of the data. When deciding what data analytics to measure, it is important that authors do not just rely on number and frequency of people using the website or app, but more what elements they engaged with and if this correlated with outcomes. This ensures that the equivalent of ‘session attendance’ is not all that is being collected. As seen in the Pham et al.^27^ scoping review, various measures were used, and although often combined, there was no standardised approach to measuring engagement with data analytics. Also, the reporting was descriptive with little emphasis on interpretation and how the findings from these measures of engagement were likely to impact on participant outcomes.^27^

As with fidelity of delivery, combining data analytics with other measures, such as participant self-reported, can provide greater insights into participants’ engagement.^28^ Such as, whether the intervention was not engaged with or whether when participants did engage, they did not find it useful so they stopped using it. However, it is important to be aware of the validity of these measures, particularly if researchers are developing them themselves. Therefore, it is of high priority to develop standardised guidelines for what constitutes as engagement and how to measure it with both data analytics and participant completed measures, to ensure the measures are valid.^28^ This becomes particularly important as self-management interventions shift from being delivered face-to-face towards being digital.

### Strengths and limitations

Due to the complex and varied nature of the interventions, it was not possible to characterise interventions and explore fidelity measures based on these and this was beyond the scope of this review. There may have been some difference between intervention types and type of measure used. In addition, within this review, we were unable to assess whether intervention fidelity was associated with interventions having significant improvements on patient outcomes, as many process evaluation papers do not report main study outcomes.

Only peer-reviewed literature was searched for studies. Due to historic publication bias, there is a likely bias against ineffective studies that were not published. As discussed earlier, it is important to conduct fidelity assessments when results are not significant to determine if the result is due to an ineffective intervention or poor implementation and/or engagement. Therefore, studies with non-significant findings should still conduct fidelity assessments and report findings to help future researchers and guideline developers make informed decisions.

### Future research and implications

To reduce burden of re-developing measures, every time a new self-management intervention is designed for a study; frameworks to measure fidelity of delivery and engagement, for both digital and face-to-face self-management interventions, should be developed. Once these measures are developed, it is essential that researchers use them to measure fidelity of intervention delivery and engagement, to have a better understanding of the active components needed to see a positive effect on clinical outcomes or to interpret non-significant findings. The measurement of fidelity should also be consistently and clearly reported by researchers, and guidelines on reporting of fidelity within trials should be developed. Therefore, future researchers investigating self-management interventions for LTCs should include at least one measure of fidelity of delivery and engagement as part of their analysis.

## Conclusion

In complex self-management interventions, it is essential to assess whether fidelity of each of the core components of the intervention was delivered, as outlined in the protocol, to understand which components are having an effect. When developing measures of fidelity of intervention delivery and engagement, the aspects of the specific intervention should be considered, for example, digital intervention versus face-to-face. Existing checklists could be built upon; however, pre-defined cut-offs for when fidelity has been achieved for each intervention must be decided. Based on the current literature, checklists for fidelity of delivery and data analytics for fidelity of engagement are the most common measures used.

## Supplemental Material

sj-docx-1-ctj-10.1177_17407745221118555 – Supplemental material for Measures of fidelity of delivery and engagement in self-management interventions: A systematic review of measures Click here for additional data file. Supplemental material, sj-docx-1-ctj-10.1177_17407745221118555 for Measures of fidelity of delivery and engagement in self-management interventions: A systematic review of measures by Tasmin A Rookes, Anette Schrag, Kate Walters and Megan Armstrong in Clinical Trials

sj-docx-2-ctj-10.1177_17407745221118555 – Supplemental material for Measures of fidelity of delivery and engagement in self-management interventions: A systematic review of measures Click here for additional data file. Supplemental material, sj-docx-2-ctj-10.1177_17407745221118555 for Measures of fidelity of delivery and engagement in self-management interventions: A systematic review of measures by Tasmin A Rookes, Anette Schrag, Kate Walters and Megan Armstrong in Clinical Trials

sj-docx-3-ctj-10.1177_17407745221118555 – Supplemental material for Measures of fidelity of delivery and engagement in self-management interventions: A systematic review of measures Click here for additional data file. Supplemental material, sj-docx-3-ctj-10.1177_17407745221118555 for Measures of fidelity of delivery and engagement in self-management interventions: A systematic review of measures by Tasmin A Rookes, Anette Schrag, Kate Walters and Megan Armstrong in Clinical Trials

sj-docx-4-ctj-10.1177_17407745221118555 – Supplemental material for Measures of fidelity of delivery and engagement in self-management interventions: A systematic review of measures Click here for additional data file. Supplemental material, sj-docx-4-ctj-10.1177_17407745221118555 for Measures of fidelity of delivery and engagement in self-management interventions: A systematic review of measures by Tasmin A Rookes, Anette Schrag, Kate Walters and Megan Armstrong in Clinical Trials
