# Supplementary material for: Measures of fidelity of delivery and engagement in self-management interventions: A systematic review of measures
Source: Clin Trials. 2022 Aug 26;19(6):665–72. doi: 10.1177/17407745221118555 (PMC9679554; doi:10.1177/17407745221118555)
Supplement: sj-docx-1-ctj-10.1177_17407745221118555 – Supplemental material for Measures of fidelity of delivery and engagement in self-management interventions: A systematic review of measures [file sj-docx-1-ctj-10.1177_17407745221118555.docx]

Supplementary Figure 1: OVID search term list example

1. Treatment fidelity, ab, ti.
2. Delivery, ab, ti.
3. Engagement, ab, ti.
4. Treatment receipt, ab, ti
5. Treatment enactment , ab, ti.
6. Program evaluation, ab, ti.
7. Process evaluation, ab, ti.
8. Implementation, ab, ti

AND

1. Self-Management/
2. self care.mp.
3. self-manag*.mp.
4. Independent Living/
