# Supplementary material for: Measures of fidelity of delivery and engagement in self-management interventions: A systematic review of measures
Source: Clin Trials. 2022 Aug 26;19(6):665–72. doi: 10.1177/17407745221118555 (PMC9679554; doi:10.1177/17407745221118555)
Supplement: sj-docx-2-ctj-10.1177_17407745221118555 – Supplemental material for Measures of fidelity of delivery and engagement in self-management interventions: A systematic review of measures [file sj-docx-2-ctj-10.1177_17407745221118555.docx]

Supplementary Figure 2. Reference list of all 39 articles included in this systematic review.

(1-39)

1. Adu MD, Malabu UH, Malau-Aduli AE, Drovandi A, Malau-Aduli BS. User Retention and Engagement With a Mobile App Intervention to Support Self-Management in Australians With Type 1 or Type 2 Diabetes (My Care Hub): Mixed Methods Study. JMIR Mhealth Uhealth. 2020;8(6):e17802.

2. Alegria M, Carson N, Flores M, Li X, Shi P, Lessios AS, et al. Activation, self-management, engagement, and retention in behavioral health care: a randomized clinical trial of the DECIDE intervention. JAMA Psychiatry. 2014;71(5):557-65.

3. Anderson JK, Turner A, Clyne W. Development and feasibility of the Help to Overcome Problems Effectively (HOPE) self-management intervention for people living with multiple sclerosis. Disabil Rehabil. 2017;39(11):1114-21.

4. Arnold C, Villagonzalo KA, Meyer D, Farhall J, Foley F, Kyrios M, et al. Predicting engagement with an online psychosocial intervention for psychosis: Exploring individual- and intervention-level predictors. Internet Interv. 2019;18:100266.

5. Aziz Z, Riddell MA, Absetz P, Brand M, Oldenburg B, Australasian Peers for Progress Diabetes Project I. Peer support to improve diabetes care: an implementation evaluation of the Australasian Peers for Progress Diabetes Program. BMC Public Health. 2018;18(1):262.

6. Battersby M, Harris M, Smith D, Reed R, Woodman R. A pragmatic randomized controlled trial of the Flinders Program of chronic condition management in community health care services. Patient Educ Couns. 2015;98(11):1367-75.

7. Ben-Zeev D, Brian RM, Jonathan G, Razzano L, Pashka N, Carpenter-Song E, et al. Mobile Health (mHealth) Versus Clinic-Based Group Intervention for People With Serious Mental Illness: A Randomized Controlled Trial. Psychiatr Serv. 2018;69(9):978-85.

8. Benzo R, Vickers K, Ernst D, Tucker S, McEvoy C, Lorig K. Development and feasibility of a self-management intervention for chronic obstructive pulmonary disease delivered with motivational interviewing strategies. J Cardiopulm Rehabil Prev. 2013;33(2):113-23.

9. Berry DL, Blonquist TM, Patel RA, Halpenny B, McReynolds J. Exposure to a patient-centered, Web-based intervention for managing cancer symptom and quality of life issues: impact on symptom distress. J Med Internet Res. 2015;17(6):e136.

10. Blonstein AC, Lv N, Camargo CA, Wilson SR, Buist AS, Rosas LG, et al. Acceptability and feasibility of the 'DASH for Asthma' intervention in a randomized controlled trial pilot study. Public Health Nutr. 2016;19(11):2049-59.

11. Busse M, Quinn L, Drew C, Kelson M, Trubey R, McEwan K, et al. Physical Activity Self-Management and Coaching Compared to Social Interaction in Huntington Disease: Results From the ENGAGE-HD Randomized, Controlled Pilot Feasibility Trial. Physical Therapy. 2017;97(6):625-39.

12. Cadilhac DA, Andrew NE, Busingye D, Cameron J, Thrift AG, Purvis T, et al. Pilot randomised clinical trial of an eHealth, self-management support intervention (iVERVE) for stroke: feasibility assessment in survivors 12-24 months post-event. Pilot Feasibility Stud. 2020;6(1):172.

13. Carron T, Bridevaux PO, Lorvall K, Parmentier R, Moix JB, Beytrison V, et al. Feasibility, acceptability and effectiveness of integrated care for COPD patients: a mixed methods evaluation of a pilot community-based programme. Swiss Med Wkly. 2017;147:w14567.

14. Crafoord MT, Fjell M, Sundberg K, Nilsson M, Langius-Eklof A. Engagement in an Interactive App for Symptom Self-Management during Treatment in Patients With Breast or Prostate Cancer: Mixed Methods Study. J Med Internet Res. 2020;22(8):e17058.

15. Feathers JT, Kieffer EC, Palmisano G, Anderson M, Janz N, Spencer MS, et al. The development, implementation, and process evaluation of the REACH Detroit Partnership's Diabetes Lifestyle Intervention. The Diabetes Educator. 2007;33(3):509-20.

16. Fraser RT, Johnson EK, Lashley S, Barber J, Chaytor N, Miller JW, et al. PACES in epilepsy: Results of a self-management randomized controlled trial. Epilepsia. 2015;56(8):1264-74.

17. Frost J, Wingham J, Britten N, Greaves C, Abraham C, Warren FC, et al. Home-based rehabilitation for heart failure with reduced ejection fraction: mixed methods process evaluation of the REACH-HF multicentre randomised controlled trial. BMJ Open. 2019;9(8):e026039.

18. Glasgow RE, Christiansen SM, Kurz D, King DK, Woolley T, Faber AJ, et al. Engagement in a diabetes self-management website: usage patterns and generalizability of program use. J Med Internet Res. 2011;13(1):e9.

19. Glasgow RE, Strycker LA, King DK, Toobert DJ, Rahm AK, Jex M, et al. Robustness of a computer-assisted diabetes self-management intervention across patient characteristics, healthcare settings, and intervention staff. Am J Manag Care. 2006;12(3):137-45.

20. Gossage-Worrall R, Hind D, Barnard-Kelly KD, Shiers D, Etherington A, Swaby L, et al. STructured lifestyle education for people WIth SchizophrEnia (STEPWISE): mixed methods process evaluation of a group-based lifestyle education programme to support weight loss in people with schizophrenia. BMC Psychiatry. 2019;19(1):358.

21. Greenwell K, Sereda M, Coulson NS, Hoare DJ. Understanding User Reactions and Interactions With an Internet-Based Intervention for Tinnitus Self-Management: Mixed-Methods Evaluation. Am J Audiol. 2019;28(3):697-713.

22. Jones F, Gage H, Drummond A, Bhalla A, Grant R, Lennon S, et al. Feasibility study of an integrated stroke self-management programme: a cluster-randomised controlled trial. BMJ Open. 2016;6(1):e008900.

23. Kelly JT, Warner MM, Conley M, Reidlinger DP, Hoffmann T, Craig J, et al. Feasibility and acceptability of telehealth coaching to promote healthy eating in chronic kidney disease: a mixed-methods process evaluation. BMJ Open. 2019;9(1):e024551.

24. Koot D, Goh PSC, Lim RSM, Tian Y, Yau TY, Tan NC, et al. A Mobile Lifestyle Management Program (GlycoLeap) for People With Type 2 Diabetes: Single-Arm Feasibility Study. JMIR Mhealth Uhealth. 2019;7(5):e12965.

25. Lambert JD, Greaves CJ, Farrand P, Price L, Haase AM, Taylor AH. Web-Based Intervention Using Behavioral Activation and Physical Activity for Adults With Depression (The eMotion Study): Pilot Randomized Controlled Trial. J Med Internet Res. 2018;20(7):e10112.

26. Leenen LAM, Wijnen BFM, van Haastregt JCM, de Kinderen RJA, Evers S, Majoie M, et al. Process evaluation of a multi-component self-management intervention for adults with epilepsy (ZMILE study). Epilepsy Behav. 2017;73:64-70.

27. Ridsdale L, Wojewodka G, Robinson EJ, Noble AJ, Morgan M, Taylor SJC, et al. The effectiveness of a group self-management education course for adults with poorly controlled epilepsy, SMILE (UK): A randomized controlled trial. Epilepsia. 2018;59(5):1048-61.

28. Ross J, Stevenson F, Dack C, Pal K, May C, Michie S, et al. Developing an implementation strategy for a digital health intervention: an example in routine healthcare. BMC Health Serv Res. 2018;18(1):794.

29. Schreurs KMG, Colland VT, Kuijer RG, de Ridder DTD, van Elderen T. Development, content, and process evaluation of a short self-management intervention in patients with chronic diseases requiring self-care behaviours. Patient Education and Counseling. 2003;51(2):133-41.

30. Sheppard DM, Gargett S, MacKenzie A, Jull G, Johnston V, Strong J, et al. Implementing a self-management intervention for people with a chronic compensable musculoskeletal injury in a workers compensation context: a process evaluation. J Occup Rehabil. 2015;25(2):412-22.

31. Shevil E, Finlayson M. Process evaluation of a self-management cognitive program for persons with multiple sclerosis. Patient Educ Couns. 2009;76(1):77-83.

32. Sinclair KA, Zamora-Kapoor A, Townsend-Ing C, McElfish PA, Kaholokula JK. Implementation outcomes of a culturally adapted diabetes self-management education intervention for Native Hawaiians and Pacific islanders. BMC Public Health. 2020;20(1):1579.

33. Steare T, O'Hanlon P, Eskinazi M, Osborn D, Lloyd-Evans B, Jones R, et al. Smartphone-delivered self-management for first-episode psychosis: the ARIES feasibility randomised controlled trial. BMJ Open. 2020;10(8):e034927.

34. Tielemans NS, Schepers VP, Visser-Meily JM, van Haastregt JC, van Veen WJ, van Stralen HE, et al. Process evaluation of the Restore4stroke Self-Management intervention 'Plan Ahead!': a stroke-specific self-management intervention. Clin Rehabil. 2016;30(12):1175-85.

35. Toomey E, Matthews J, Hurley DA. Using mixed methods to assess fidelity of delivery and its influencing factors in a complex self-management intervention for people with osteoarthritis and low back pain. BMJ Open. 2017;7(8):e015452.

36. Verwey R, van der Weegen S, Spreeuwenberg M, Tange H, van der Weijden T, de Witte L. Process evaluation of physical activity counselling with and without the use of mobile technology: A mixed methods study. Int J Nurs Stud. 2016;53:3-16.

37. Wilde MH, Liebel D, Fairbanks E, Wilson P, Lash M, Shah S, et al. A hybrid process fidelity assessment in a home-based randomized clinical trial. Home Health Care Serv Q. 2015;34(2):113-36.

38. Wilson M, Roll JM, Corbett C, Barbosa-Leiker C. Empowering Patients with Persistent Pain Using an Internet-based Self-Management Program. Pain Manag Nurs. 2015;16(4):503-14.

39. Zinken KM, Cradock S, Skinner TC. Analysis System for Self-Efficacy Training (ASSET). Assessing treatment fidelity of self-management interventions. Patient Educ Couns. 2008;72(2):186-93.
