# Supplementary material for: Measures of fidelity of delivery and engagement in self-management interventions: A systematic review of measures
Source: Clin Trials. 2022 Aug 26;19(6):665–72. doi: 10.1177/17407745221118555 (PMC9679554; doi:10.1177/17407745221118555)
Supplement: sj-docx-4-ctj-10.1177_17407745221118555 – Supplemental material for Measures of fidelity of delivery and engagement in self-management interventions: A systematic review of measures [file sj-docx-4-ctj-10.1177_17407745221118555.docx]

Supplementary Table 2. Full details of the 39 studies included in the review, including author, intervention name and aim, long term condition, what was delivered, how and when, and reporting of fidelity of delivery and engagement.

| **Lead author, year, and country** | **Intervention name** | **Participant long term condition** | **Aim of intervention** | **What was provided and delivered** | **Intervention leader Method and location of delivery** | **When and how much** | **Fidelity of delivery** | **Fidelity of Engagement** |
| --- | --- | --- | --- | --- | --- | --- | --- | --- |
| Adu, 2020, Australia | My Care Hub | Type 1 and 2 Diabetes | Improving patients’ awareness about diabetes distress and potential ways to reduce its impact on their self-management | Access to the app 1. Documentation of clinical measures 2. Analytics displaying visual graphs 3. Diabetes education | Digital intervention App Remote | 3 weeks Could log in as much as wanted | No | Yes |
| Alegria, 2013, USA | DECIDE (Decide the problem; Explore the questions; Closed or open-ended questions; Identify the who, why, or how of the problem; Direct questions to your health care professional; Enjoy a shared solution) | Mental Health Conditions | Teaches patients to  1. Identify decisions regarding their behavioural healthcare 2. Generate questions for their healthcare professionals regarding these decisions 3. Promote interactions with healthcare professionals | Training sessions that present information whilst encouraging participation, role-play and reflection.  1. Decisions and agency 2. Role, Process and Reason 3. Self-Efficacy and Consolidation | Care Managers Face-to-face  Community outpatient mental health clinics | 3 sessions of 30-45 minutes each over a 3-month period.  Booster session was offered, if necessary. | Yes | No |
| Anderson, 2017, UK | Help to Overcome Problems Effectively (HOPE) | Multiple Sclerosis | Manualised self-management intervention combining positive psychology theory and practice, and cognitive behavioural therapy | Group sessions focussed on psychoeducation, skills practice, discussion and goal setting, with homework tasks | Lay leaders with MS Face-to-Face MS Society branch | 6-weekly sessions lasting 2.5 hours each. | Yes | No |
| Arnold, 2019, Australia | Self-Management and Recovery Technology (SMART) | Psychosis | A resource on self-management and personal recovery | Access to the SMART website supplemented with weekly, alongside emails from an online coach over a 12-week period | Digital intervention and online coach Web-program Remote | 12 weeks Could log in as much as wanted | No | Yes |
| Aziz, 2018, Australia | Peers for Progress Diabetes Program PfP-DP | Type 2 Diabetes | Peer support functions:  1. Assistance in daily health management 2. Social and emotional support  3. Promotion of links to clinical care and community resources  4. Ongoing support to assist with diabetes self-management | Monthly group meetings for social and emotional support, goal setting, educational resources including diabetes education booklets and DVDs | Lay leaders with type 2 diabetes Face-to-Face Local community authorities | 12 monthly sessions over 12 months | Yes | No |
| Battersby, 2015, Australia | Flinders Program | COPD, Coronary artery disease, cerebrovascular disease, chronic heart failure, diabetes or musculoskeletal disorders | Based on cognitive, behavioural, and motivational theory and practice and uses a set of tools, the Partners in Health Scale, Cue and Response Interview, and life Problem and Goals assessment to complete an integrated self-management and medical care plan | 12-month care plan tailored to individuals’ problems and goals.  One-to-one sessions to monitor progress | Health worker Face-to-Face Patient's home or at a health service Or on the telephone | Every 2-4 weeks for 6 months totally 5 hours of contact. | Yes | No |
| Ben-Zeev, 2018, USA | mHealth FOCUS app | schizophrenia, schizoaffective disorder, bipolar disorder, or major depressive disorder | Content targets 5 domains: 1. Auditory hallucinations 2. Mood 3. Sleep 4. Social functioning 5. Medication | FOCUS application (app), clinician dashboard, and mHealth support specialist, with self-assessment prompts Content is video or audio clips or text. | Digital intervention App Remote | Functions can be accessed 24 hours per day | No | Yes |
| Benzo, 2013, USA | Combined self-management education with motivational interviewing | COPD | Self-management education and motivational interviewing, to facilitate the resolution of ambivalence, fostering engagement in self-management, and encouraging behaviour change. | Learn key behaviours for COPD managements and then complete self-management action planning | Nurse and therapist Face-to-Face  Hospital clinic | 8 weekly sessions totally 4.5 hours | No | Yes |
| Berry, 2015, USA | Electronic Self Report Assessment-Cancer (ESRA-C) | Cancer | Supports patients with cancer during treatment to increase the frequency of patient-clinician communication, reduce symptom distress, and increase the patient’s unsolicited and specific description of symptoms and quality of life concerns | Following the quality-of-life report, the intervention group participants received pushed teaching tips for the QoL issues reported as moderate-to-severe, which could be expanded addressing  (1) “Why does this happen?” (2) “What can I do about this?” (3) “What do I tell my clinical team?” | Digital Intervention Web-program Clinic or Patient's Home | Could access the program at any time from home or on a screen in the clinic | No | Yes |
| Blonstein, 2015, USA | DASH for asthma | Asthma | Focussed on dietary modifications and smoking cessation underpinned by Social Cognitive Theory | Topics included nutrition information and behaviour modification strategies, with an emphasis on sustainable changes to eating habits and practices. Participants received a binder to which handouts, worksheets and home activity checklists. | Lifestyle coaches (dieticians) Face-to-Face and Remote Clinic and Telephone | 3 individual and 8 group sessions in the first 3 months, followed by telephone consultations | Yes | Yes |
| Busse, 2017, UK | Physical Activity Self-Management and Coaching (ENGAGE-HD) | Huntington Disease | Grounded in self-determination theory: 1. Participant/coach interaction 2. Engage-HD Workbook 3. Exercise DVD (Move to Exercise) | Developed up to three realistic physical activity goals and were assisted with individual physical activity progression through goal discussion. Exercise diaries and pedometers were provided | Therapists Face-to-Face and Remote Patients Home and Telephone | 6 home visits and 3 phone calls over 14 weeks | Yes | No |
| Cadilhac, 2020, Australia | iVERVE | Stroke | Enables the programming of electronic support and educational messages aligned to nominated goals, based on behaviour change theory and Information-Motivational- Behavioural theory | A comprehensive post-discharge support eHealth program that was delivered via the iVERVE system. Received daily support messages matched to their goals and ability | Healthcare professional Remote Telephone and Messages | 1 text a day for 4 weeks | Yes | No |
| Carron, 2017, Switzerland | Living Well with COPD: A Plan of Action for Life | COPD | Patient education and self-management, scheduled follow-up, multidisciplinary care co-ordination, training of healthcare professional’s ad evidence-based COPD care | Disease education, medication, breathlessness and stress management, prevention and management of exacerbations, and lifestyle behaviours, as well as an action plan | Respiratory physiotherapist and/or nurse specialist Face-to-face in clinic and telephone | 6 weekly group sessions last 90-120 minutes each and 2 one-to-one sessions.  Followed by monthly calls for the next 12 months | Yes | No |
| Crafoord, 2020, Sweden | Interaktor app | Breast or Prostate Cancer | Symptom assessment, an alert system for contact with health care professionals, access to self-care advice, and visualization of symptom history | Reporting of symptoms on days when receiving chemotherapy / radiotherapy treatment and continue for 2-3 weeks after | Digital intervention Remote App | 9-12 weeks access to the app | No | Yes |
| Fraser, 2015, USA | PACES in Epilepsy | Epilepsy | Emphasis on the range of mood and cognitive concerns across all modules, as well as education and emotional coping strategies | PACES workbook, SMART goal setting alongside topics: Epilepsy and Medical Issues; Dealing with Stress and The Blues; Optimizing My Cognition; Community Participation on a Budget; Managing My Epilepsy Care; Assertive Communication; and My Health and Well-Being. | Rehabilitation psychologist and peer leader  Face-to-Face | 8 weekly 75-minute sessions | No | Yes |
| Frost, 2018, UK | REACH-HF | Heart Failure | A comprehensive selfcare support programme comprising the ‘REACH-HF Manual,’ which includes lay explanations of HF and how people can learn to live with the condition to maximise their quality of life | Manual combined with exercise programmes, information about managing medications, monitoring symptoms, and managing stress, anxiety and low mood. Plus, a progress tracker and a resource for carers. | Facilitators  Face-to-face and remote Home and telephone | 4-6 one to one sessions | Yes | No |
| Glasgow, 2006, USA | The Diabetes Health Connection | Diabetes | In-person, computer-assisted behaviour change program to facilitate healthful dietary and physical activity practices | A health behaviour assessment and an education program via computer with tailored self-management sessions with feedback, goal setting around diet and exercise, and strength training plan | Health Connection coaches | 30–40-minute session, with2 follow-up phone calls | Yes | No |
| Glasgow, 2011, USA | Computer-Assisted Self-management: “My Path to Healthy Life” | Diabetes | Applies behaviour change principles at patient, health care provider, and social-environmental levels, drawn from social-cognitive theory and self-efficacy and application of social-ecologic approaches to health issues | Access to an Internet-based website with information about medication adherence, exercise and food choices, as well as setting and monitoring achievable goals to enhance self-efficacy in these 3 areas. In another group participants also received 2 follow-up calls for social support | Digital intervention and diabetes care manager Remote Online and Telephone | Access to the website as much as needed and text messages every 6-10 weeks.  Two 10-minute phone calls in social support group | No | Yes |
| Gossage-Worrall, 2019, UK | STructured lifestyle education for people WIth SchizophrEnia (STEPWISE) | Schizophrenia, schizoaffective disorder or first episode psychosis | Involves interactions between healthcare professionals, service users and equipment. Focus on resources, activities, and immediate, intermediate and long-term outcomes | Each session covered how diet and activity lifestyle changes could help participants take control of their weight. Participants shared their stories and helped with problem solving and a healthy lunch was provided | Healthcare professional Face-to-face and remote Clinic and telephone | 12 sessions over 12 months lasting 2.5 hours each 10-minute telephone every 2 weeks | Yes | No |
| Greenwell, 2019, UK | Tinnitus E-Programme | Tinnitus | Information resources cover the mechanisms of tinnitus, stress and its management, attention focus, and negative thinking. Several behaviours change techniques are used to promote relaxation behaviour | (a) education resources about tinnitus and its management; (b) training/rehearsal for psychological strategies; (c) online discussion forum to provide social support; (d) self-monitoring of tinnitus distress; and (e) information about available resources | Digital intervention Remote Online | 6 weekly online sessions with 4 weeks of relaxation goal setting | No | Yes |
| Jones, 2016, UK | Bridges Self-Management Programme | Stroke | Seven principles: 1. Problem solving 2. Reflection 3. Goal setting 4. Accessing resources 5. Self-discovery 6. Activity 7. Knowledge | 1. One-to-one rehabilitation sessions to support self-management 2. A workbook that included vignettes, activities, ideas and solutions from other stroke survivors for successful self-management, and space to record and reflect on goals | Healthcare professionals Face-to-Face Community Stroke Rehabilitation Services | Unclear | Yes | No |
| Kelly, 2019, Australia | ENTICE-CKD programme | Chronic Kidney Disease | Social cognitive theory, with a patient-centred focus on improving self-management to reduce dietary sodium intake and increase dietary quality. The constructs of social cognitive theory most used were outcome expectation, self-regulation and self-efficacy | Workbook: setting SMART goals; eating well for kidneys; active living; role of diet in kidney disease, strategies for planning, self-monitoring checklists and a list of useful websites, apps and recipes. Telephone call content was guided by the workbook topics, structured according to the 5A’s framework (Assess, Advise, Agree, Assist, Arrange), and tailored to participants. | Telehealth Coach (dietician) Remote Telephone | One telephone call per fortnight (30-45 mins) and 2–8 tailored text messages for 3 months, and then 4–12 tailored text messages for 3 months without telephone calls | Yes | Yes |
| Koot, 2019, Singapore | GlycoLeap | Type 2 Diabetes | Educational curriculum delivered through online lessons and the Glyco mobile phone app with a health coaching feature, enabling users to log and monitor their blood glucose levels, weight, meals, and physical activity. Dietitians provide personalized feedback to participants on their progress and to present opportunities for improvement. | Gluco app, glucometer kit with lancets and test strips, a BodyTracewireless weighing scale, diet and activity educational guidebooks, and a resistance band were provided | Digital intervention Remote App | 24 weeks of unlimited access to Glyco app | No | Yes |
| Lambert, 2018, UK | eMotion | Depression | Based on behavioural activation with added physical activity | Weekly web-based course, with 13 modules, teaching daily routines, increasing activities through positive reinforcement. | Digital intervention Remote Website | 13 modules over 8 weeks, minimum dose were the first 2 modules. | No | Yes |
| Leenen, 2017, The Netherlands | ZMILE study | Epilepsy | A multi-component self-management intervention based on managing symptoms and physical and psychosocial consequences through self-monitoring and risk management, with an emphasis on proactive coping, shared decision-making, and goal setting | The sessions consist of education and practicing goal-setting skills, covering self-monitoring; risk evaluation; and shared decision-making / concordance to enhance self-efficacy by discussing and sharing strategies. Also, two eHealth tools were used for self-monitoring | Facilitators Face-to-Face Clinic | Five weekly group sessions of 2 h each, followed by a two-hour booster session after three weeks. | Yes | No |
| Ridsdale, 2018, UK | SMILE | Epilepsy | The premise of the course was to communicate information and to encourage participants to share their own experiences with others. | Participants were given a workbook containing course content to use during the sessions and to take home. | Nurse specialist Face-to-Face Community | 2 consecutive days of 8-hour sessions | Yes | No |
| Ross, 2018, UK | HeLP-Diabetes (Healthy Living for People with Type 2 Diabetes) | Type 2 Diabetes | Online programme, addressing patient needs including education, lifestyle changes, medicine management, emotional management, and social support, to be used alongside existing face-to-face programmes e.g., DESMOND | An online programme that can be accessed on desktop computers, tablets, and mobile devices with an internet connection | Digital intervention Remote Online | Unlimited access to online programme | No | Yes |
| Schreurs, 2002, The Netherlands | Self-management intervention | Asthma, Diabetes and Heart Failure | Proactive coping: 1. Resource accumulation 2. Attention and recognition  3. Potential stressors are appraised 4. Preliminary coping  5. Feedback elicited and used | Sessions started with discussing homework and then introducing the session topic: self-monitoring; recognising and acting on early symptoms; medication and adaptations; lifestyle recommendations; disease specific tasks. Their beliefs, emotions, experiences and barriers were discussed, and an action plan developed. A workbook was provided | Specialist nurses Face-to-Face Clinic | Five 2-hour group sessions | No | Yes |
| Sheppard, 2015, Australia | Self-Management for Return to Work (SMRTW) | Musculoskeletal Disorder | Based on CDSMP, which aims to enhance skills and self-efficacy to allow more confident management of the chronic condition. | The six-week program featured small group dynamics and skill attainment including problem solving and goal setting. Two additional modules focusing on participants’ knowledge and self-efficacy in navigating injury compensation systems were also available | Lay leader and vocational rehabilitation (VR) consultant Face-to-face Community | Eight weekly 2-hour sessions | Yes | Yes |
| Shevil, 2009, US | Mind over Matter | Multiple Sclerosis | Problem-solving therapy techniques taught participants how to identify daily tasks that are cognitively challenging, generate potential solutions, and apply cognitive-management strategies to perform the tasks. The multicontext approach guided the choice of strategies and their application to promote transfer and generalization. | 1. Increase knowledge of cognitive symptoms and their impact on daily functioning 2. Increase levels of self-efficacy in ability to self-manage cognitive symptoms 3. Enhance ability to self-manage cognitive symptoms through the use of cognitive solutions and strategies | Occupational Therapist Face-to-face and telephone Community | 5 weekly 2-hour group sessions | Yes | No |
| Sinclair, 2020, US | Partners in Care | Diabetes | Focus group themes included multi-level difficulties in adherence to behaviours, increased physical activity, and lack of knowledge about the importance of glucose control. These were combined with feedback from a community-academic steering committee, peer educators and evidence based clinical guidelines and Social Cognitive Theory | Educational sessions with additional incentives were provided: a pill box during Lesson 2: Medicine and Glucose Balance; a stretch band during Lesson 5: Move More, Sit Less!; and a colander to rinse and drain fat in Lesson 7: Diabetes and Cholesterol | Peer educator Face-to-face Community | 12 weekly 1-hour sessions | Yes | No |
| Steare, 2019, UK | App to support Recovery In Early intervention Services (ARIES) | Psychosis | The aim of the intervention is to develop users’ self-management skills to help them to achieve self-determined recovery goals and avoid future relapses. | My Journey 3 app incorporates self-management to allow users to track recovery goals and personalise relapse prevention plans | Digital intervention Remote App | One 2-hour training session followed by unlimited access to the app for 12 months | No | Yes |
| Tielemans, 2016, The Netherlands | Restore4stroke Self-Management intervention ‘Plan Ahead!’ | Stroke | Aims to increase stroke patients’ participation in activities by teaching proactive action planning through 4 themes: 1. handling negative emotions 2. social relations and support 3. participation in society 4. less visible consequences of stroke | Asked questions that focus on thinking in terms of goals, opportunities and solutions, instead of problems and barriers and motivated participants to work on their proactive action plans. | Rehabilitation professionals Face-to-Face Outpatient Clinic | 10-week programme with six 2-hour sessions and a 2-hour booster session in the last week | Yes | No |
| Toomey, 2016, Ireland | Self-Management of Osteoarthritis and Low back pain through Activities and Skills (SOLAS) | Osteoarthritis and low back pain | To promote self-management through group discussion on targeted self-management skills or behaviours, whilst setting and reviewing goals and developing action plans | Sessions split into 45-minute exercise and education component with 4 categories:  1. Materials  2. Introduction and review 3. Education 4. Review and planning | Physiotherapist Face-to-Face Community | 6 weekly group sessions lasting 90 minutes | Yes | No |
| Two Feathers, 2007, USA | REACH Detroit Partnership’s Diabetes Lifestyle Intervention | Diabetes | Social cognitive theory with an emphasis on behavioural capability, self-control procedures, emotional coping response, and self-efficacy | Provision of healthy snacks, delivery of meeting content and activities, goal setting, and anonymous participant feedback | Family Health Advocates Face-to-Face Community | Five 3-hour group meetings delivered every 4 weeks | Yes | Yes |
| Verway, 2016, The Netherlands | Self-management Support Programme with an embedded tool | Type 2 Diabetes and COPD | The consultations were based on the ‘Five A’s cycle’ counselling technique (Assess–Advise–Agree–Assist– Arrange) | Information booklet about the intervention and locally organised physical activities. Homework set between sessions 1. Activity diary 2. Activity plan 3. Review progress Tool included an app linked to an activity monitor showing live activity results | Practice nurse and Digital intervention Face-to-Face and Remote GP Practice and App | Four consultations over 4-6 months | Yes | No |
| Wilde, 2015, USA | Self-care management intervention | Catheter users | Nurse, home visits to help patients monitor urine flow to improve self-management of indwelling urinary catheters and reduce untoward outcomes | Teaching self-management skills through urinary diary and education booklet. | Nurse Face-to-face Home and Telephone | 3 home visits and 1 telephone call over 4 months | Yes | No |
| Wilson, 2015, USA | Chronic Pain Management Program (CPMP) | Persistent Pain | A self-directed, self-paced Internet-based self-management program intended for a general population of patients with persistent noncancer pain | Generates an individualized custom plan and summary report based on the results of the Profile of Chronic Pain assessment for pain intensity, pain interference, and emotional burden to guide treatment planning. PCP scores map onto learning modules that fall into four categories: cognitive, behavioural, social, and emotional regulation | Digital intervention Remote Website | 8-week access to the online programme | Yes | Yes |
| Zinken, 2008, UK | Group-based self-management programme | Type 1 Diabetes | Social Cognitive theory | Alongside diabetes specific issues such as matching insulin and food, and managing hypoglycaemia, enhancement of personal confidence was a key component of the intervention | Facilitators Face-to-Face Clinic | 6 weekly sessions lasting 3 hours | Yes | No |

Supplementary Table 3. Full details of the measures of fidelity of intervention delivery for the 25 studies measuring it, including what was measured, types of measure, who completed the measure, how it was developed, how it was analysed and the findings.

| **Author (year)** | **What was measured?** | **Types of measures?** | **Who completed the measures?** | **Development of measures** | **Sample data captured for** | **Analysis method** | **Summary of findings** |
| --- | --- | --- | --- | --- | --- | --- | --- |
| Alegria, 2013, USA | Adherence to intervention manual | A 50-item checklist, reflecting the essential components of trainings | Unclear | Unclear | Random sample of the recorded trainings of 45 patients | Descriptive statistics | Adherence was 87% for training 1, 84% for training 2, and 60% for training 3. |
| Anderson, 2017, UK | Intervention quality and delivery | 9 item scale: The health Education Impact Questionnaire (HEIQ) scored: 1=Strongly Disagree, 2=Disagree, 3=Agee, 4=Strongly Agree | Participants | Nolte et al. (2007) | All participants | Descriptive statistics | The mean ratings for all 9 items were 3.6/4 or above, indicating that participants rated the intervention delivery and quality positively. |
| Aziz, 2018, Australia | Implementation | Using RE-AIM (Reach, Effectiveness, Adoption, Implementation, Maintenance Using PIPE (Penetration, Implementation, Participation, Effectiveness) | Researchers | Glasgow et al (1999) and Pronk (2003) | All participants | Descriptive statistics | Implementation coefficient for: peer leaders training (95%), weekly teleconference (91%), and Monthly group meetings (92%); making a total of 93% for the study. Adoption coefficient: setting-level adoption 11/12 - 92% Reach coefficient: 273/441 - 62% Effectiveness coefficient: 65% |
|  |  | Questionnaire | Participants | Designed by researchers | All participants | Descriptive statistics | 56% reported no barriers. 44% reported one or more factors including health issues (37%), lack of time/work commitments (37%), location/timings (37%), and issues in family/family commitments (14%). Most participants stated that they intended to continue to use strategies learnt in this program including healthy diet (91%), seeking timely clinical care (82%), regular exercise (77%), blood glucose monitoring (76%), and utilising social and emotional support (74%). |
| Battersby, 2015, Australia | Quality of delivery | Scoring of 'goals' for compliance with training manual | Independent raters | Compared to manual | All participants receiving intervention | Descriptive statistics | 66% of the behavioural goals available for assessment were consistent with the requirements in the manual. |
| Blonstein, 2015, USA | Diary completion, topics covered and quality | Quantitative questionnaires and random sample of 10% of recorded sessions were rated for quality using a 'standard rating scale' | Participants and lifestyle coaches | Unclear | All participants who received intervention | Descriptive Statistics | In general, most sessions were conducted per protocol. Among a total of 116 individual sessions, 106 covered all planned topics, 3 sessions did not cover one topic and 1 session did not cover any of the planned topics. Of 40 group sessions, 37 covered all planned topics, 2 sessions did not cover one topic and 1 session had missing data. 96% completed the food diary.  Overall quality and adherence to script of individual and group sessions and overall quality of telephone sessions were high. For individual and group sessions combined, mean score of overall quality was 4.6/5 adherence to script was 14.1/15. For telephone sessions, mean score of overall quality was 4.2/5. |
| Busse, 2017, UK | Fidelity | Self-report checklists, independent analysis of audio recordings and self-assessment | Coaches and researchers | Unclear | 15/16 of 3rd intervention session | Descriptive statistics | Overall scores ranged from 7-14 out of 16, with a mean (SD) score across the coaches of 11.0 (2.4). Coach interactions scored an average of 2.5/4 for autonomy, 3.0/4 for relatedness, 2.7/4 for competence, and 2.8/4 for the overall impression. Self-assessment scores were on average higher than those assigned by the independent rater, namely 3.1/4 for autonomy, 3.3/4 for relatedness, and 3.0/4 for competence. |
| Cadilhac, 2020, Australia | Intervention fidelity | Number of text messages sent, those failed to send, number of respondents who sent ‘STOP’ texts and the reasons for the ‘STOP’ texts | N/A | N/A | All those in intervention group | Descriptive statistics | There were no message failures and no calls to the help desk to report problems with the messages. In terms of returned messages, there were two ‘STOP’ requests |
| Carron, 2017, Switzerland | Fidelity | Comparing the protocol to what had been done, as described in annual reports | Unclear | Unclear | Unclear | Unclear | Good adherence to the programme protocol, but some adaptations were needed to respond to local constraints and the sessions could only take place in 3 out of the 4 areas. |
| Feathers, 2007, USA | (1) fidelity to the curriculum, (2) participants’ questions, (3) information added by FHAs, and (4) observed participant understanding and satisfaction with content and activities | A checklist of critical intervention behaviours and the material delivered during each meeting.  A written questionnaire | Family health advocates | Unclear | Unclear | Descriptive statistics | Based on observations, the FHAs delivered 97% or more of the curriculum material and 90% of the activities. 98.4% expressed satisfaction with the intervention meetings. In response to the question, “What information did you find especially useful?” 97% of respondents cited specific meeting content and activities that were useful, including label reading, stress-reducing techniques, role-playing, group, and individual problem solving, and goal setting. |
| Frost, 2018, UK | Fidelity | 13-domain checklist to assess facilitators' delivery of the intervention. Fidelity scoring attributed a numerical value (0–6) for each of the domains. Fidelity of intervention was adequate if scored ≥3 for each domain. | Researchers | Developed and piloted during the feasibility study | 110 / 332 contacts (34%) | Descriptive statistics | Fidelity was adequate (score of ≥3 out of 6) in most domains. There was room for improvement, particularly with respect to involving the caregiver, addressing caregiver concerns, and addressing caregiver well-being, where the mean scores were well below the agreed ‘adequate’ score. |
| Glasgow, 2006, USA | Implementation - Fidelity aspects of the RE-AIM framework | Implementation: percentage of patients who received each of the key treatment elements | Researchers | RE-AIM framework | All those in intervention group | Descriptive statistics | The intervention was implemented consistently by all intervention staff, and there were no differences in the application of the 3 implementation variables. All participated in the computer-assisted program and reviewed their plan with their interventionist. Follow-up telephone calls were completed with 96% of the TSM group subjects. |
| Gossage-Worrall, 2019, UK | Fidelity (Design, training, delivery, receipt, enactment) | Direct observation of sessions using the STEPWISE Core Facilitator Behavioural Observation Sheet (CFBOS) testing presence or absence of 35 behaviour domains. The DESMOND Observation Tool (DOT) also measured facilitator versus service user talk time using timed audio cues during a sample of sessions. | Researchers | From other trial papers - unclear which ones | All participants in intervention arm | Descriptive statistics | Fidelity assessment showed mean (SD) facilitator talk time was 47.6% (12.2%) and facilitator behaviours deemed positive was 54.1% (17.6).  Lapses in fidelity were observed by assessors e.g., giving answers, rather than eliciting, solutions from service users to discussion topics. All foundation and all but one booster sessions were delivered as scheduled. |
| Jones, 2016, UK | Fidelity | A checklist to record patient and professional activities and behaviours against each principal component of the SMP. | Researchers | Piloted to enable a method to compare self management support delivered in intervention and control sites that could be used in a larger trial | A proportion of rehabilitation sessions - n=14 | Descriptive statistics | The checklist was feasible to use and identified whether CSR incorporated behaviours and activities relating to core self-management principles. Clinicians in the intervention sites showed use of between five and seven self management principles, whereas those in the control site showed evidence of using two or less. |
| Kelly, 2019, Australia | Intervention delivery | All coaching calls were audio recorded, from which 10% were assessed for consistency (the predefined call scripts and potential deviation from the call scripts with reasons why) | Peer-review by an individual external to the project | Developed by researchers rather than validated surveys | All participants in intervention arm | Descriptive statistics | 90% of planned calls being completed according to the protocol |
|  |  | Number, duration, and content of coaching telephone calls; number and type of text messages delivered; number and type of text message responses; and time spent by coaches for each interaction |  |  |  |  | The mean duration of the first intervention call was 45.5±10 min and the subsequent five calls was 24±10 min. A total of 4985 text messages were sent, with a median of four text messages per fortnight in phase 1 and seven per fortnight in phase 2 per participant. The total number of replies from participants was 1100, 36% triggered the appropriate goal-check reply, 3% required the dietitian coach to send a tailored text message and 61% required no reply. |
| Leenen, 2017, The Netherlands | Participant’s evaluation of the intervention | 1) perceived usefulness of the meetings and the eHealth tools on a 7-point Likert scale; 2) their opinion on seven statements about the purpose and content of the intervention (1 = totally disagree–7 = totally agree) | All participants and relatives who attended at least 1 group session | Unclear | 47 participants and 32 carers | Descriptive statistics | >74% rated the sessions as useful but thought they would be more educational. |
|  | Protocol violations | Unclear | Facilitators | Compared to protocol |  |  | Every intervention group received the planned 6 sessions, which lasted for 2 h. The mean group size was 8.1. 46/47 patients and 30/32 relatives formulated an action plan and most stated they were still using it. |
| Risdale, 2018, UK | Implementation | Intervention delivered according to the protocol | Unclear | Unclear | Unclear | Unclear | Trainers within the trial delivered the intervention according to protocol, with good adherence and high competence. |
| Sheppard, 2015, Australia | Implementation | Researcher notes and program leaders’ evaluation/feedback forms | Program leaders' | Unclear | All participants in intervention arm | Descriptive statistics | Unable to recruit and retain participants, so changed from Phase 3 RCT to Phase 2 feasibility study. The content was delivered as intended. |
| Shevil, 2009, US | Delivery | The facilitator completed reflection notes following each session. A standard template was used to develop each note and addressed attendance, adequacy of allotted time to cover material, completion of homework, additional comments raised by participants, and general issues about session delivery. | Facilitators | Unclear | All participants in intervention arm | Descriptive statistics | Unclear |
| Sinclair, 2020, US | Fidelity | Checklist of critical intervention behaviours and materials to be delivered during each class to monitor: 1) fidelity to the curriculum, 2) participants’ questions, 3) information added by peer educators, 4) research staff’s overall impressions of participant understanding and satisfaction. | Researchers | Rated on a scale of 1 to 4/5 (e.g., did not follow curriculum at all to followed all the curriculum / no one understood to all understood / not satisfied at all to very satisfied) | 12 randomly selected classes | Descriptive statistics | Intervention fidelity was rated as high (4.8 on a scale from 1 to 5), indicating that peer educators delivered at least 98% of the curriculum material as written and conducted 95% of the proposed activities. Activities that weren't conducted were due to time constraints. Impressions of understanding of was rated high (4.7 on a scale from 1 to 5), and satisfaction with content and activities was 3.8 on a scale from 1 to 4, indicating high satisfaction. |
| Tielemans, 2016, The Netherlands | Dose received | Engagement from session log | Therapist | Unclear | All participants who attended at least 1 session (n=53/58) | Descriptive statistics | In 20% of the sessions, the proactive action plan tool was inadequately used according to the therapist sessions logs. No group was assessed below sufficient or acceptable levels, and most groups were assessed to perform beyond these levels. 47% worked on their goals during all five sessions requiring goal setting, 23% did all the homework assignments with a decline noticed after session 4. |
| Toomey, 2016, Ireland | Fidelity | A priori checklist against the intervention manual. | Researchers | Toomey et al., 2016 fidelity protocol | 40% of sessions were observed, 100% audio recordings | Mean difference with ANOVA and Kruskal-Wallis and Wilcoxon test | Fidelity was high in all assessment methods, with a mean score of 81.7% for the audio-recordings, 82.7% for the direct observations.  All methods found a statistically significant difference between the actual duration of the exercise component and its intended duration of 45 min for session 1. |
| Verway, 2016, The Netherlands | Exposure to the interventions | Record of all consultations in log files Data from the online tool / app | Nurses | N/A | Participants in the tool / app arm of the study | Descriptive statistics | 2/3rds of sessions went on for longer than planned.  The 5 A's model was mostly executed as planned (60-90%).  Many reported that goals were not discussed or set.  98% of participants registered with the app, 81% completed 'preparation for target setting', 63% set up an activity plan and 83% read their feedback messages |
| Wilde, 2015, USA | Intervention delivery | Tape recordings or direct observation, looking at adherence to protocols and nurse competence on a 5-point scale of performance from 1 (not at all), 2 (a little), (somewhat), 4 (considerably), to 5 (extensively). | Independent raters | Unclear | 10% of the 300 home visits were selected at random | Descriptive statistics | Most ratings were well over 4 on the 5-point scale for adherence and competence, with a slight increase in scores over time. There was little evidence of variability between nurses. |
|  |  | Checklist and assessments about presence or absence of barriers and participant issues/concerns that affected the nurses’ ability to complete all planned components as scheduled and/or that impacted the patient’s ability to follow up with the self-management teaching. | Nurses | Unclear | All participants who in intervention arm | Descriptive statistics and bivariate correlates | External barriers to participation included a lack of support from family and friends and transportation issues.  Internal barriers included lack of motivation, comorbidities, and lack of confidence.  There was a positive relationship between level of participant interest with diary completion (p=.022) and progress towards goal (p=.001). |
| Wilson, 2015, USA | Progress towards goal | Questionnaire - 4-item Likert scale (0= ‘‘no progress’’; 4= ‘‘met goal’’) | Participants | Unclear | All participants who in intervention arm = 92 | Descriptive statistics and chi squared | 81% reported some progress towards medication goals, in comparison to 49% in control (chi^2 = 5.77, p = .02). 76% reported some progress towards health and wellbeing goals, in comparison to 69% in control (chi^2 = 0.37, p = .54). |
| Zinken, 2008, UK | Inter-rater reliability | Cohen's Kappa with a score ranging from 0 to 1. Counted the frequencies and recorded the duration of each specific technique that occurred within the programme | Trained coders | Literature review and observing 2 interventions to identify techniques for enhancement of self-efficacy, used to develop the coding criteria. | Audio recordings of all 12 group sessions | t-test chi square ANOVA | The reliability measured with Cohen’s Kappa was good. The agreement was 0.71 and 0.70 for super and subcategories, respectively.  Less time was spent devoted to the enhancement of self-efficacy and as the programme progressed less time was left for the participant to talk and talking about self-efficacy (r = -0.91, p < 0.05), contrary to how the programme was designed to be implemented. |

Supplementary Table 4. Full details of the engagement measures for the 19 studies measuring it, including what was measured, types of measure, who completed the measure, how it was developed, how it was analysed and the findings.

| **Author (year)** | **What was measured?** | **Types of measures?** | **Who completed the measures?** | **Development of measures** | **Sample data captured for** | **Analysis method** | **Summary of findings** |
| --- | --- | --- | --- | --- | --- | --- | --- |
| Adu, 2020, Australia | Participants’ app usage log | App use (number of active users, frequency of daily access to app), data logs/time spent, and number of opened notifications. | N/A | N/A | All participants receiving intervention | Descriptive statistics | Authors compared their engagement findings to other similar studies and rated frequency usage rate as reasonable, which was initially high but decreased in following weeks and suggested push notification could further stimulate users’ engagement |
|  | Verbal feedback | The FITT index: 1. Frequency index (F): how often participants use the app, return to the app and active app users 2. Intensity index (I): proportion of users who interact with each feature in the app: (1) frequency of daily use of app (2) number of notifications opened  3. Type index (T): Form of engagement based on actions performed using the available features.  4. Time index (T): the duration of engagement, as daily duration with each feature. | N/A | Authors adapted the Frequency, Intensity, Time, and Type (FITT) principal index |  |  | The active time spent on the documentation features demonstrated that the duration of app usage necessary to generate consistency is a parameter that depends on individual users. The app system was unable to capture whether participants were reading and comprehending the embedded information or simply clicking them. An approach to address this limitation is to incorporate eye-tracking technology or tailored quizzes into My Care Hub to measure cognitive responses and knowledge acquired through engagement with each information screen. |
| Arnold, 2019, Australia | Engagement / usage | Logins and activities i.e., watching videos, listening to audio content, external links, peer forum, completing exercises, viewing topic pages | N/A | Within website | All participants receiving intervention | Descriptive statistics and analytics Mann-Whitney U | Over the 12-week intervention period, all participants logged in to the website a total of 985 times and completed 4698 activities. ‘Recovery’ was the most viewed module, with 543 activities completed. ‘Life’ was the least viewed module, with 101 activities completed. The most common type of activity was “view website content or sections” (N=1527). The chart feature was also used frequently with 526 total entries. Forum posts received many views (N=427), but few comments (N=88): 46 participants viewed the forum, but only 18 participants contributed. Tertiary education was the only predictor of engagement for depth of use (U=853.0, P=.036). |
| Benzo, 2013, USA | Fidelity | Goal set, confidence | Participants | Scales including the Working Alliance Inventory—Short Revised | All participants receiving intervention | Descriptive statistics | Goals were set in 83% of sessions. Patients reported that (1) they frequently agreed with the interventionist about strategies to improve the patient situation (“very often” or “always” 88.5%), (2) they had confidence that the interventionist could help them (“very often” or “always” 84%), (3) they worked toward mutually agreed-upon goals (“very often” or “always” 84.6%) and agreed on what was important to work on (“very often” or “always” 84.6%), and (4) the way they were working on their problems was correct (“very often” or “always” 100%). |
| Ben-Zeev, 2018, USA | Engagement | Weekly use of FOCUS app (engaged if used 5/7 days a week) | By app | N/A | All participants | Descriptive statistics | 90% used the app at least once and on average 5.4 days in the first week, 4.6 days in the third week, 4.3 days in the sixth week, 3.9 days in the ninth week, and 3.8 days in the last week. |
| Berry, 2015, USA | Exposure | (1) the number of pushed teaching tips during prompted sessions, (2) the number of clicks on the non-pushed Teaching Tips tab during prompted and non-prompted sessions, and (3) the number of clicks on the View My Reports tab during prompted and non-prompted sessions. | N/A | Within app | All participants receiving intervention | Descriptive statistics | 62.3% of participants used the intervention at least once.  34% of participants voluntarily used the intervention.  Software error meant full exposure could not be recorded for all participants. |
| Blonstein, 2015, USA | Engagement and understanding | rated on a 5-point Likert scale after each session | Coaches | Unclear | All participants receiving intervention | Descriptive statistics | Level of participant engagement rated as 4.4 / 5; level of participants understanding rated as 4.5 / 5 for each session. Both were 0.1 higher for individual over group sessions. |
| Crafoord, 2020, Sweden | Engagement | Logged data on app, including symptom reports, triggered alerts, views of self-care advice, and free text messages sent | N/A | N/A | All participants receiving intervention | Descriptive statistics | All patients reported with the app at least once. Median adherence was 83% and remained stable over time, although there was a drop towards the end.  100% of breast cancer group viewed self-care advice at least once and viewed a median of 11/17 self-care advice topics at least once. 87% of the prostate cancer group viewed self-care advice at least once and the median number of self-care advice topics viewed at least once was 5/16. Most patients used the free text function at least once: 93% in the breast cancer and 75% in the prostate cancer group. |
| Feathers, 2007, USA | Participant engagement in sessions | Researcher notes / observation | Researchers | Unclear | All participants | Descriptive statistics | Participants appeared to enjoy the curriculum materials and activities and interacted with FHAs and other participants. They frequently asked questions, engaged in discussion, and shared their own stories related to the meeting topic and struggles with controlling their diabetes. |
| Fraser, 2015, USA | Goal attainment summary | Ranked on a five-point Likert scale, ranging from “much less than expected” (1) through “much greater than expected” (5), and a “3” rating being “expected level” of goal attainment. | Participants | Unclear | All participants receiving intervention | Descriptive statistics | The average number of goals set was 7.6 and the average achievement rating was "slightly higher than expected" 3.19. Over time fewer goals were set after week 6 out of 8. |
| Glasgow, 2011, USA | Website usage and engagement | Automated data - mean number of visits, median number of visits, and the percentage of patients who visited each section of the website at least twice, number of action plans created, time spent on the site | N/A | N/A | All participants receiving intervention | Descriptive statistics | Participants demonstrated large variability in website usage over the 4 months, ranging from 1 to 119 website visits (mean 28 visits). Usage decreased over time, with 70% visiting at least weekly during the first 6 weeks and 47% during weeks 7 to 16. Total time on website averaged a little over 3 hours or about 7 minutes per visit. 75% used the tracking feature at least once a week and on average 1.7 out of 3 action plans were completed. |
| Greenwell, 2019, UK | Engagement | Use of intervention. | Participants | Unclear | First 50 people to respond who had used the intervention - only 27 analysed | Descriptive statistics | 11 participants used the complete intervention, 10 used some (including the two health professionals), 1 used only the forum, and 6 did not use the intervention. Participants with tinnitus who used “some” of the intervention reported using at least some of the information leaflets and relaxation exercises. Under half joined the forum and read posts. |
| Kelly, 2019, Australia | Intervention Adherence | Adherence to the dietary intervention, collected by coaches in each telephone call measured as: evidence of the participant’s overall progress, evidence of self-monitoring, goals set and implementation intentions. | Participants | Developed by researchers rather than validated surveys | All participants | Chi square test | Participants set new goals with 26% updating at least one goal in call two and 61% updating at least one goal throughout the remaining four calls. 76% of participants showed evidence of self-monitoring by the second call, which was sustained. 82% of participants needed at least 2 calls to begin putting planned dietary intentions in place. |
| Koot, 2019, Singapore | Program Engagement | Data analytics from app use and logging of information in the app | N/A | Within app | All participants | Descriptive statistics | Generally high participant engagement was observed for all components in the first week which then decreased progressively over time. On average, participants finished 9.2 lessons. Fewer than 20 participants logged their blood glucose measurements at least 4 times a week yet more than 25 participants logged their weight measurements at least once a week. On average, participants entered 2.1 meal logs and sent 2.8 messages to their health coach each week |
|  |  |  |  |  |  | Per-protocol linear regression | Linear regression showed HbA1c decreased by an average of 1.0 percentage point more among those who logged their weight more (P=.007). No statistically significant associations found between change in weight and program engagement. |
| Lambert, 2018, UK | Participant receipt and enactment | Web based questionnaires - “I understand how emotions, behaviours, thoughts, and physical feelings affect each other to maintain depression over time.” And rating their confidence in using specific BCTs | Participants | A single item based on questions from a previous study adapted from similar measures of BCT usage, providing initial evidence of the validity of this type of measure | 46 participants | Adjusted mean difference | Those in eMotion group more likely to report significant different on confidence scale than control |
|  | Quality / completeness of delivery | Website usage statistics from the Web-based intervention database | N/A | N/A | All participants | Descriptive statistics | Overall, 53% (17/32) of participants completed at least the introduction, week 1 and 2, minimum dose. |
| Ross, 2018, UK | Usage data | Data analytics from website i.e., people signing up at each GP |  | Within app | All GP practices signposting to the website | Descriptive statistics | If no patients being signed up, there may be a problem with implementation. This monitoring prompted contact with staff in these GPs and for feedback from staff to be collected. |
| Schreurs, 2002, The Netherlands | Evaluation of the intervention | Evaluations of sessions and the course were elicited by written statements which participants agreed on a 5 point-scale ranging from (1) ‘not at all’ to (5) ‘very much’. Time spent on homework and frequency of doing homework assignments. | Participants | Aggregated statements into scales. 2) evaluation of homework assignments 3) evaluation of proactive coping by goal setting and making action plans | 66/83 participants who completed the course | Descriptive statistics | Homework assignments were rated less positively than the usefulness of goal-setting strategies and action-planning (t (57) = 3:98, P < 0.01). Homework assignments were generally done on 3–4 days per week, and patients spent almost 20 min on each. |
| Sheppard, 2015, Australia | Dose Received | Attendance | Researchers | Unclear | Participants who met the attendance criteria 5/8 sessions N=17 | Descriptive statistics | The program leaders, observing each session, noted that participants participated in key components of the program and that it took 3–4 weeks before participants identified and attained achievable goals. |
| Steare, 2019, UK | Acceptability and engagement | App usage data: including each time the user opened My Journey 3, whether this was in response to a prompt and which component they used. |  |  | All participants receiving intervention | Descriptive statistics | All participants attended training session and had access to My Journey 3 during the trial. The median number of times My Journey 3 was used per participant during the trial was 16.5. Participants accessed My Journey 3 on a median of 3.22% of the days it was available to them, equating to My Journey 3 being used on average once every 31 days. Participants spent a median of 26.8 min in total using the app throughout the trial. 25% were still using the app 6 months after downloading it. 1 person never used it. |
| Wilson, 2015, USA | Engagement | Star Ratings' - An engagement level of ‘‘0’’ was assigned if there was no evidence of activity within any learning module; a ‘‘1’’ was assigned if there was no evidence of activity within any learning modules but the PCP was completed; and so on, up to the highest level of ‘‘5’’ assigned if all four activities showed evidence of participation. | N/A | Created when participants exit a learning activity in the CPMP | All participants receiving intervention | Descriptive statistics | 38% engaged in at least half of the CPMP learning modules, within these modules engagement in activities varied from 1 to 21 activities completed. No evidence that those more engaged had better outcomes. |
